# Retained Austenite Decomposition and Carbide Precipitation during Isothermal Tempering of a Medium-Carbon Low-Alloy Bainitic Steel

**DOI:** 10.3390/ma11081441

**Published:** 2018-08-15

**Authors:** Seyyed Hesamodin Talebi, Mohammad Jahazi, Haikouhi Melkonyan

**Affiliations:** 1 Département de Génie Mécanique, École de Technologie Supérieure, Montréal, QC H3C 1K3, Canada; 2 Finkl Steel Inc., 100 McCarthy, Saint-Joseph-de-Sorel, QC J3R 3M8, Canada; hmelkonyan@finkl.com

**Keywords:** tempering, dilatometry, retained austenite, chromium carbide, transmission electron microscopy (TEM)

## Abstract

The effect of isothermal tempering on retained austenite decomposition and carbide precipitation were investigated in a medium-carbon low-alloy bainitic steel. High-resolution dilatometry was used to perform isothermal tempering at 350 °C, 550 °C and 600 °C for different holding times up to 16 h. The decomposition of retained austenite, morphology and composition of carbides were investigated by analyzing the dilatometric curves and were confirmed through scanning and transmission electron microscopy observations. The decomposition behavior of retained austenite varied significantly as a function of the tempering temperature with a full decomposition observed at 600 °C. It was also found that by increasing the tempering temperature from 550 °C to 600 °C, carbides precipitate approximately twice as fast, and evolve from M_3_C type to Cr_7_C_3_ and Cr_23_C_6_ after 16 h of tempering at 600 °C.

## 1. Introduction

Medium-carbon, low-alloy steels containing chromium and nickel are used as mold material for the production of plastic components employed in transportation industries [1,2,3]. Following ingot casting, these steels undergo open die forging and quench and temper operations to achieve the required sizes and mechanical properties, respectively. In recent years, with the growing industrial demand for ever larger parts, the forged blocks have significantly increased in size. Therefore, during the quench process, significant temperature gradients are produced that result in a variety of microstructures, such as martensite, bainite and retained austenite through the thickness of the large-size block [4,5]. The hard martensitic phase is generally located near the surface region with the proportions of bainite and retained austenite increasing with the thickness [6]. Such heterogeneity in the microstructure translates into variable mechanical properties from one location to another of the block and could impact the machinability and service durability of the mold.

The objective of the tempering process is to reduce or eliminate the undesirable retained austenite and improve the ductility of the material by decreasing the carbon supersaturation in the martensitic and bainitic constituents through carbide precipitation [6,7]. Carbon segregation, precipitation of transition carbides, retained austenite decomposition, carbide precipitation and carbide coarsening are the main microstructural changes that take place during the tempering process [8,9,10,11].

It is known that during cooling of austenite and formation of bainite, the reaction stops when the carbon content of retained austenite is equal to the composition specified by the T_0_ curve, instead of the equilibrium A_3_ curve [12]. Therefore, the mixed microstructure composed of carbon-enriched retained austenite and bainite is not stable from a thermodynamic point of view [12]. Accordingly, keeping this microstructure at an elevated temperature for adequate time should result in the decomposition of the retained austenite. The product of retained austenite decomposition, depending on steel composition, can be ferrite and carbides, martensite or bainite, which affect the mechanical properties [13,14]. Therefore, the determination of the kinetics of retained austenite decomposition is of significant practical as well as theoretical importance. In addition to retained austenite decomposition, the type and size of carbides that precipitate during tempering affect mechanical properties and therefore need to be engineered during the tempering process [15,16,17]. Five types of carbides, M_3_C, M_2_C, M_7_C_3_, M_23_C_6_ and M_6_C, have the potential to precipitate in medium carbon low alloy steels, based on tempering time and temperature [18,19,20,21]. Among them, M_23_C_6_ is the equilibrium carbide, M_3_C is the stable carbide, M_2_C and M_7_C_3_ are metastable carbides [18,19,22,23].

While tempering of quenched steels has been extensively studied; however, little data is available on isothermal tempering of low-alloy bainitic mold steels, particularly, on the determination of the tempering temperature required for complete retained austenite decomposition, and identification of the types of carbides that can form during tempering of these steels. The present work has been defined in this framework and the objective is to investigate the influence of tempering holding time and temperature on carbon-enriched retained austenite decomposition and the formation of carbides, using a combination of high resolution dilatometry, and scanning and transmission electron microscopies.

## 2. Materials and Methods 

The chemical composition of the studied steel is provided in Table 1.

**Figure 1:**
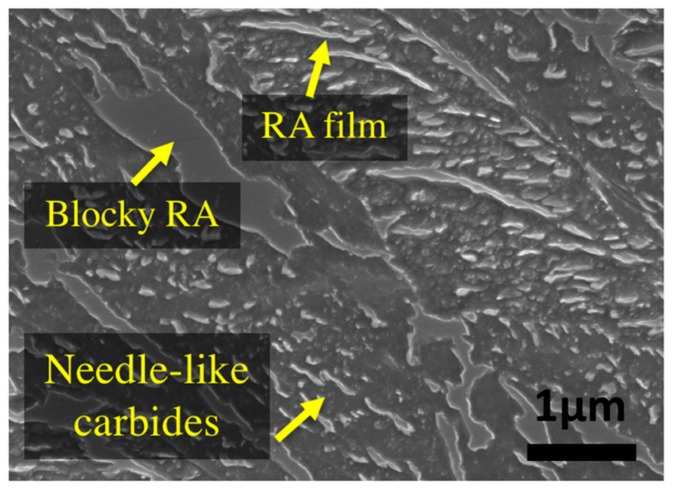
Primary microstructure of bainite with retained austenite before tempering.

The original material was an as-forged slab with initial dimensions of 6000 mm × 1200 mm × 800 mm, provided by Finkl Steel Inc. (Sorel, QC, Canada). Initially, a 127 mm diameter and 177 mm length ‘carrot’ was extracted from a region about 200 mm below the surface (quarter of the thickness). Cylindrical samples of 10 mm length and 4 mm diameter were machined from the ‘carrot’ for dilatometry experiments. All the heat treatment cycles were performed using a high-resolution TA DIL 805A/D dilatometer (TA instruments, New Castle, DE, USA). Prior to the tempering experiments, the bainitic specimens were austenitized by heating them up to 870 °C, held for 10 min for homogenization and then cooled to room temperature with a cooling rate of 4.8 °C/min which produced a microstructure composed of bainite and about 23% of retained austenite. Figure 1 shows the primary bainitic microstructure before isothermal tempering. Examination of the microstructure revealed the presence of needle-like carbides, thin films, and blocks of retained austenite at the grain boundaries of the ferritic bainite which are characterized by a smooth surface under SEM (Scanning Electron Microscope). Chemical analysis of the carbides before tempering indicated that they are very rich in Fe with nearly no presence of Cr.

Isothermal tempering at three temperatures of 350 °C, 550 °C and 600 °C with different holding times up to 16 h were performed immediately after cooling. Samples were heated with a heating rate of 600 °C/min up to isothermal tempering temperature. The fast heating rate was used in order to avoid any effect of heating rate on microstructure change during heating. The tests were performed under vacuum to avoid any oxidation and decarburization. To study the microstructure, after dilatometry tests, samples were cut, using very thin diamond blades, in the longitudinal direction. They were then prepared through conventional polishing procedures and etched with Vilella solution. Retained austenite measurement was carried out according to ASTM E975 using a Bruker Discover D8-2D diffractometer (Bruker, Madison, WI, USA) with Co Kα radiation in standard θ–2θ mode. A Hitachi-SU8230 Field Emission Gun SEM (FEG-SEM) (Hitachi, Tokyo, Japan) was used to study the microstructures. TEM observations were made by a JEOL JEM-2100F transmission electron microscope (JEOL, Tokyo, Japan) equipped with Selected Area Electron Diffraction Analysis (SAED) operated at 200 kV acceleration voltage on carbon extraction replicas. Average Rockwell C hardness of each sample was measured from 121 tests under loading conditions of 2 kg for 10 s.

## 3. Results and Discussion

### 3.1. Retained Austenite Decomposition 

**Figure 2:**
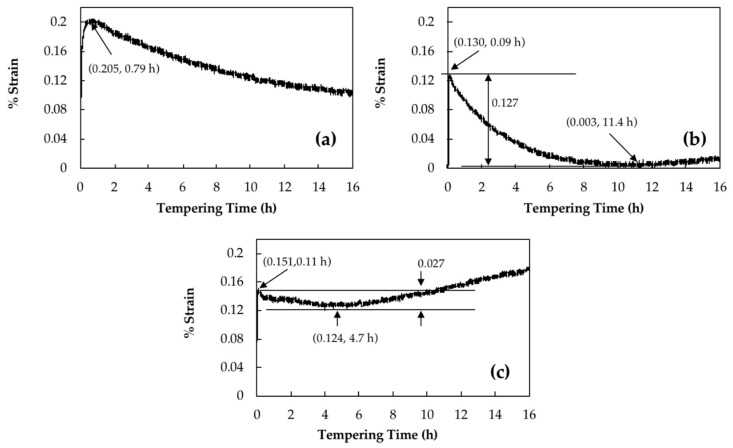
Dilatometry curves during isothermal tempering for 16 h at (**a**) 350 °C; (**b**) 550 °C and (**c**) 600 °C.

Retained austenite decomposition should be accompanied by a length increase of the sample [16]. Curves in Figure 2a–c show the strain changes of dilatation curves versus tempering time for 16 h of isothermal tempering for a bainitic specimen containing 23% of retained austenite at 350 °C, 550 °C and 600 °C, respectively.

For instance, it can be seen in Figure 2a that by tempering at 350 °C, strain increased sharply and reached a maximum amount of 0.20% after 0.79 h, then slowly decreased without reaching a plateau even after 16 h. Podder et al. [13] and Yan et al. [24] observed similar behavior in a bainitic structure and related it to the start of the decomposition of retained austenite. Since 350 °C is the lowest temperature for retained austenite decomposition [6], the maximum amount of increase due to retained austenite decomposition is about 0.20%.

However, tempering at higher temperature causes carbide precipitation, which is characterized with a length reduction. This is illustrated in Figure 2b, where at 550 °C a 0.13% of length increase is observed during the first 0.09 h of tempering and after that, the specimen’s length decreased until 11.4 h of tempering when it reached its initial length before tempering. During tempering at 600 °C, 0.02% more length increase occurred in comparison with 550 °C, which was followed by around 0.03% length decline until 4.7 h of tempering; afterwards, remarkable length increase was observed (Figure 2c). Therefore, the samples tempered at 600 °C and 550 °C show only an increase of 0.15% and 0.13%, respectively. It should be noted that the higher expansion observed during the early stages of tempering at 600 °C indicates that a higher amount of retained austenite has been decomposed at this temperature compared to 550 °C.

The possible presence of retained austenite in the microstructure was investigated using X-ray diffraction (XRD). However, no retained austenite peak was detected. Considering that the minimum detection limit by XRD is 5%, it can be said that the amount of retained austenite in the tempered microstructure is less than 5%. Subsequently, high-resolution dilatometry, which can reveal the presence of retained austenite at much lower levels and its transformation during the tempering cycle was conducted [8,24].

**Figure 3:**
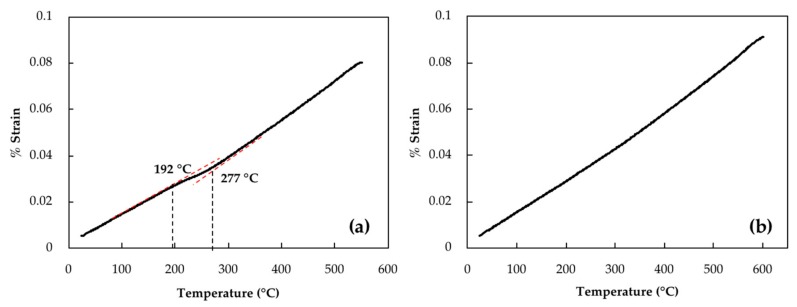
Dilatometry curves of cooling cycle with cooling rate of 600 °C/min after isothermal tempering for 16 h at (**a**) 550 °C and (**b**) 600 °C.

Retained austenite which remains undecomposed during tempering can be transformed to fresh martensite during cooling to room temperature [13]. To investigate any possible martensitic transformation during cooling of the investigated steel, samples were cooled with a cooling rate of 600 °C/min. Figure 3a,b illustrate dilatometry curves of cooling after 16 h of tempering at 550 °C and 600 °C, respectively. In Figure 3a, a transformation point can be seen that starts at 277 °C and finishes at 192 °C. These two points are associated with martensite start (M_s_) and finish (M_f_) temperatures. Similar martensitic transformations were observed after tempering at 550 °C for 1 h, 4 h, 8 h and 12 h with M_s_ values of 228 °C, 256 °C, 260 °C and 272 °C, respectively. However, examination of the dilatometry curve at 600 °C, reported in Figure 3b, did not reveal any length changes related to martensitic transformation, indicating that there is very low or no more retained austenite to be transformed into martensite. Therefore, it can be said that isothermal tempering of 16 h at 600 °C has decomposed any detectable retained austenite. The decomposition is characterized on the dilatometry curve by a 0.15% increase in the strain.

**Figure 4:**
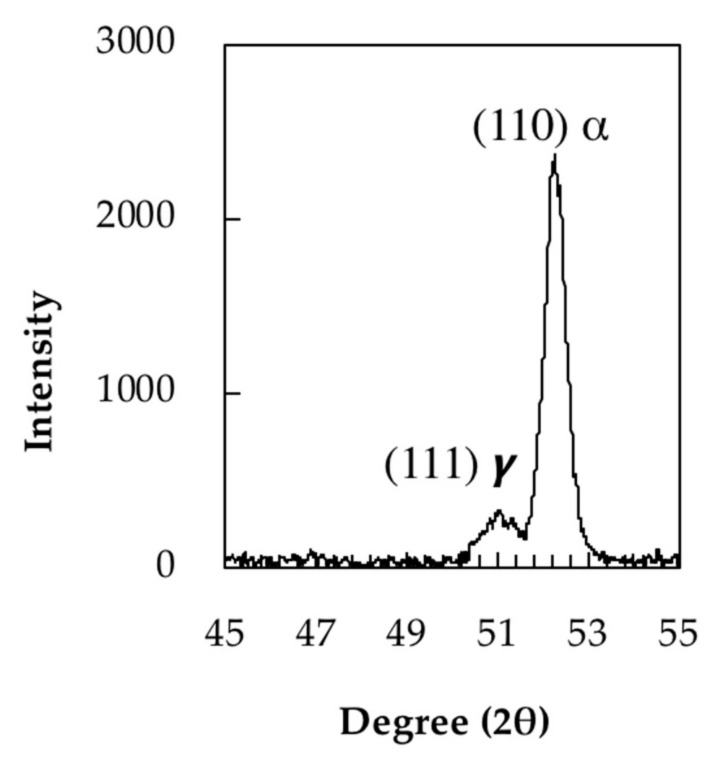
XRD (111) peak of retained austenite.

The carbon content of retained austenite before tempering was determined by XRD from lattice parameter measurements. Figure 4 shows the (111) peak of retained austenite. The lattice parameter (*α*_0_ *γ*) measured for this austenite is 0.35801 nm. The carbon content of the retained austenite was estimated to be 0.24 wt.% based on the following Equation (1) [25,26]:

(1) $$a_{0}\;\gamma =3.572+0.033\left(wt.\%\;C\right)$$

**Figure 5:**
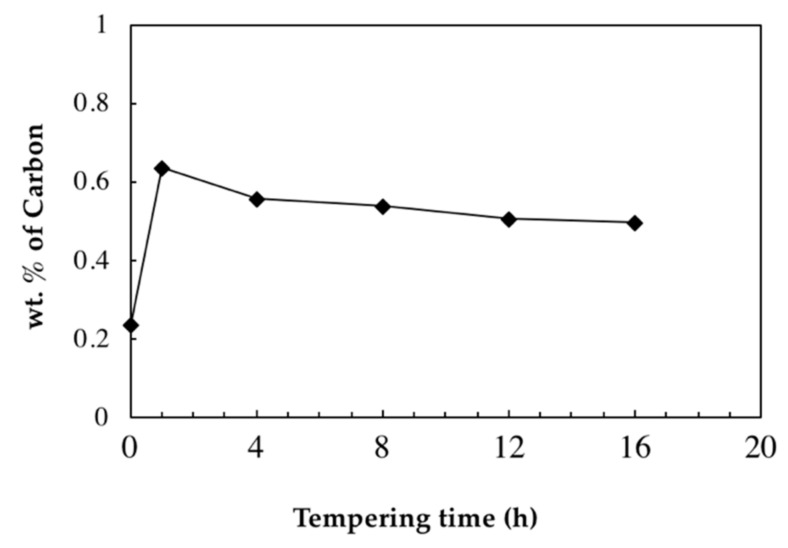
Change in carbon content of retained austenite during tempering at 550 °C.

Based on the M_s_ temperatures determined by the slope change of the cooling curves and using the following Equation (2) [27], the carbon content of retained austenite was calculated after different holding times at 550 °C and the results are reported in Figure 5:

(2) $$Ms\;\left(K\right)=273+545.8\cdot e^{-1.362w_{C}}$$

where, $w_{C}$ represents the weight percentage of carbon in austenite.

It can be seen from Figure 5 that the carbon content of retained austenite which is 0.24 wt.% before tempering becomes 0.64 wt.% after 1 h of tempering at 550 °C due to the decomposition of retained austenite. This amount continuously drops during tempering until reaching 0.50 wt.% after 16 h of tempering. The above results agree with the findings by Bhadeshia [12], who reported that austenite to bainite transformation continues until the amount of carbon reaches the amount defined by the T_0_ curve (i.e., retained austenite is expected to become enriched in carbon with the tempering time as long as the decomposition continues).

The fact that the carbon content of retained austenite has been reduced during isothermal tempering demonstrates that the decomposition of retained austenite did not occur during the entire tempering process and ceased, or significantly slowed down, during the first hour of isothermal tempering at 550 °C, where carbon content of retained austenite dropped from 0.64% after 1 h to 0.56% after 4 h.

### 3.2. Products of Retained Austenite Decomposition

**Figure 6:**
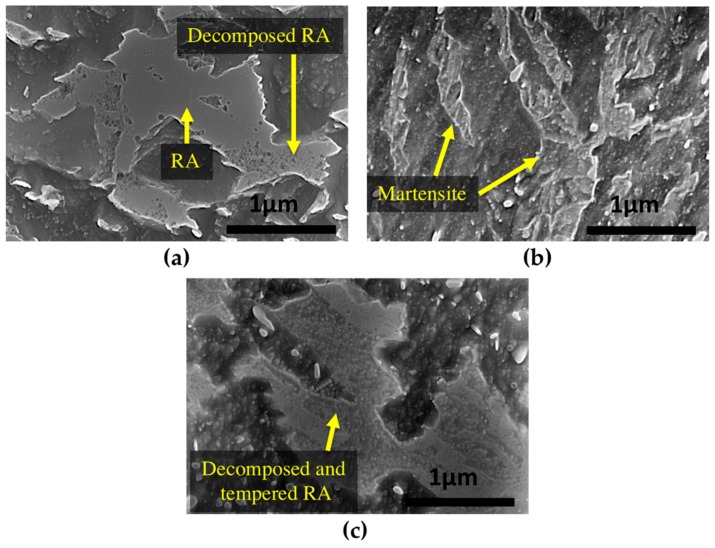
SEM images of (**a**) blocks of partially decomposed retained austenite after 16 h of tempering at 350 °C; (**b**) fresh martensite formed during cooling after 16 h of tempering at 550 °C and (**c**) fully decomposed retained austenite and tempered microstructure blocks after 16 h of tempering at 600 °C.

During tempering, retained austenite transforms to lower bainite. Figure 6a shows that at 350 °C there are still many untransformed islands of retained austenite, indicating that this temperature is not high enough for its complete transformation. In contrast, as shown in Figure 6b, after 16 h of tempering at 550 °C, fresh martensite can be seen, instead of decomposed austenite, which confirms martensite formation in the microstructure during the cooling cycle.

The final product of retained austenite decomposition includes precipitation of new M_3_C carbides. During tempering at 600 °C, these new carbides rapidly spheroidize [19] and as shown in Figure 6c, the product of tempered retained austenite includes small spherical-shape carbides inside the previous blocky type retained austenite.

### 3.3. Carbides Precipitation and Hardness Evolution

Carbide precipitation during tempering is characterized by a length reduction in the dilatometry diagram [8,9]. The obtained results in the present investigation are in agreement with the published literature and as shown in Figure 2, a reduction in length is observed for the three samples. Further tempering causes carbide coarsening, which is accompanied with a length increase as is shown in Figure 2b,c. The presence of a negative strain after retained austenite decomposition, as observed in Figure 2a, demonstrated that when tempering at 350 °C, the precipitation of carbides did not finish even after 16 h. Carbon trapping in lower energy sites, such as dislocations has been reported as the main source for the observed slow length reduction during tempering [28,29].

**Figure 7:**
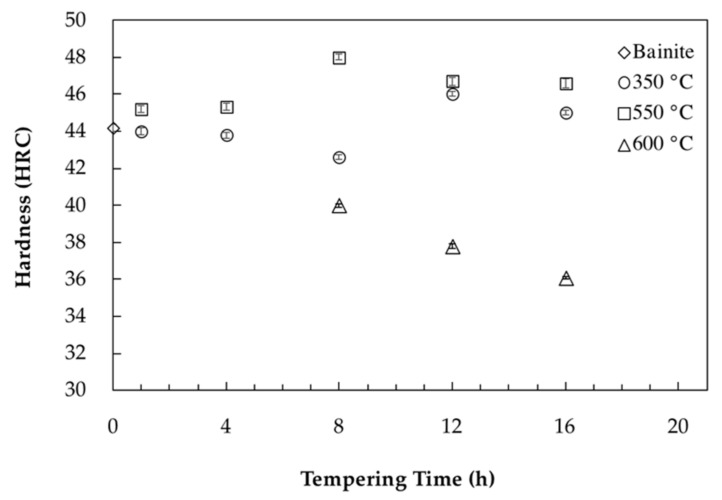
Bainite hardness as a function of tempering time for tempering temperatures of 350 °C, 550 °C and 600 °C.

In order to better illustrate the impact of the tempering process on the evolution of mechanical properties, Figure 7 shows hardness evolution as a function of different tempering times and temperatures. In the case of tempering at 350 °C, as shown in Figure 7, minor variations (between 42 HRC and 46 HRC) were observed by increasing the time. This fluctuation is probably due to the partial decomposition of retained austenite into fresh bainite and its tempering during holding.

During tempering at 550 °C, carbides precipitation caused a 0.127% decrease in length and end after 11.4 h of tempering (Figure 2b). This behavior correlates well with hardness evolution, which increased up to 8 h of tempering at 550 °C and reached the high value of 48 HRC. Analysis of the dilatometry curves indicated that after 11.4 h of tempering, carbide coarsening characterized with a positive strain value is the dominant mechanism which causes a marginal drop in hardness from 48 HRC to 46.5 HRC.

Carbides precipitation during tempering at 600 °C ended after 4.7 h tempering with a 0.027% length reduction observed in the dilatometry curves. The lower length reduction compared to 550 °C is probably due to more intense carbide coarsening kinetics (characterized with higher length increase) occurring simultaneously with carbide precipitation during tempering at 600 °C. Comparison of hardness values between the two tempering temperatures of 550 °C and 600 °C, demonstrates that 50 °C increase in tempering temperature leads to a significant reduction in the time required for carbide precipitation and modification of the hardness levels. As shown in Figure 7, for the sample tempered 8 h at 600 °C, carbide coarsening drops the hardness to 40 HRC and further tempering up to 16 h decreases the hardness to 36 HRC. Accordingly, hardness results are consistent with dilatometric curves and the sequence of carbide precipitation and coarsening show that carbide coarsening occurs after 4.7 h of tempering at 600 °C (Figure 2c).

### 3.4. Characterization and Identification of Carbides

**Figure 8:**
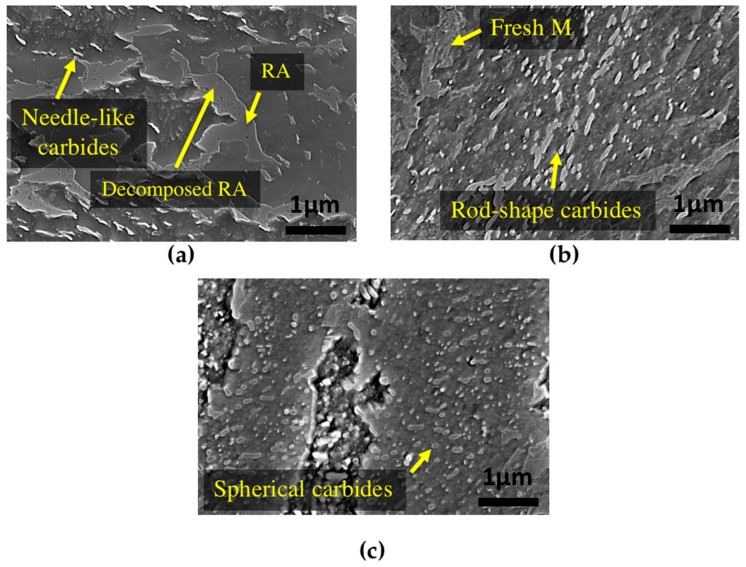
SEM images of bainitic specimens after tempering for 16 h at (**a**) 350 °C; (**b**) 550 °C and (**c**) 600 °C.

SEM analysis was performed before and after tempering to confirm and validate the dilatometry results. Figure 8a illustrates that tempering at 350 °C leads only to partial changes in the microstructure. Similar to the primary bainite microstructure (Figure 1), needle-like carbides can still be seen after 16 h of tempering at this temperature, and there is no obvious change in the shape of carbides and bainite.

The microstructure of the sample tempered after 16 h at 550 °C is shown in Figure 8b, where a severely tempered bainitic microstructure can be observed. In this specimen, needle-like carbides within the bainite sheaves are transformed to coarser rod-shape carbides. Fresh martensite can also be observed, whose presence is the result of retained austenite transformation to martensite during cooling after tempering. In comparison with tempering at 550 °C, increasing the temperature to 600 °C with the same tempering time led to major morphological changes in the bainite (Figure 8c). Although still some rod-shape carbides can be seen in the microstructure, the morphology consists of globular shape carbides with no needle-like carbide.

During tempering of bainitic steels containing chromium at temperatures higher than 500 °C, Cr_7_C_3_ and Cr_23_C_6_ chromium carbides are frequently observed, which form according to the following sequence [30]:

(3) $$Matrix\;\to \;Fe_{3}C\;\to \;Cr_{7}C_{3}\;\to \;Cr_{23}C_{6}$$

**Figure 9:**
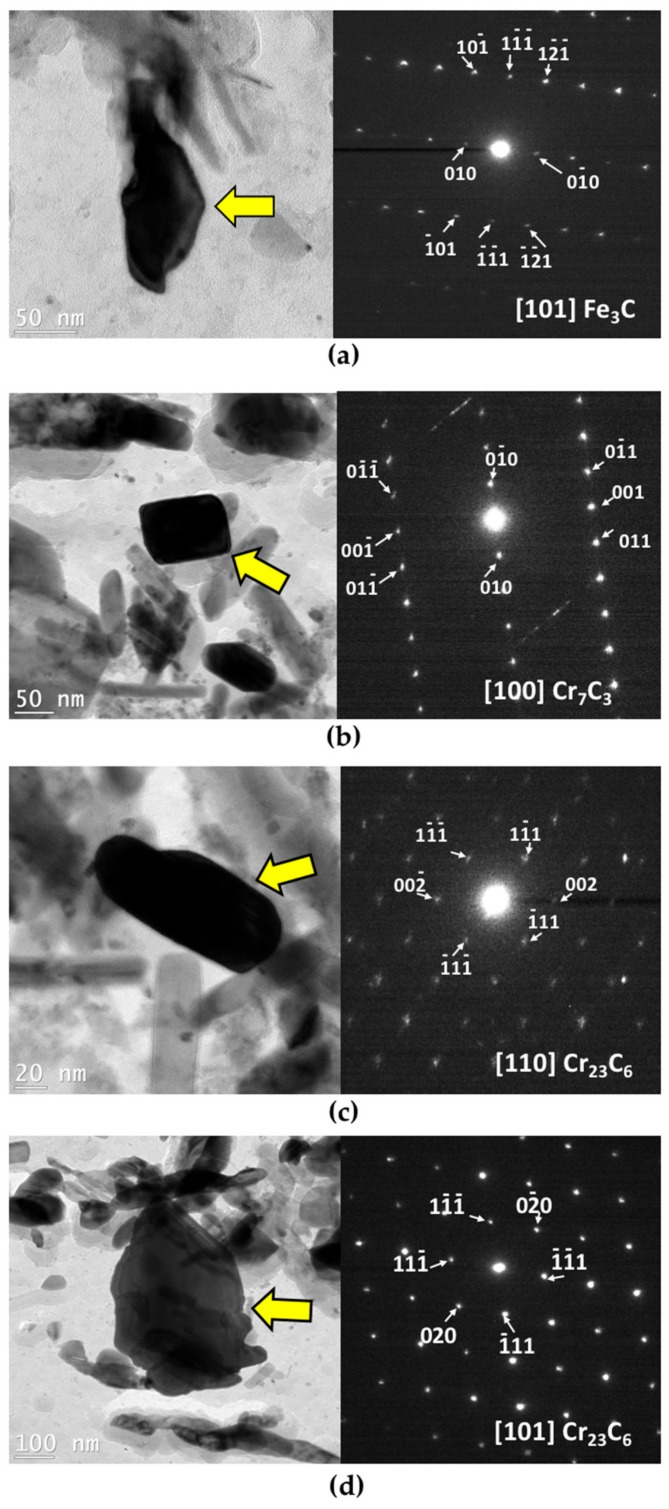
Bright-field TEM image and corresponding selected area electron diffraction (SAED) pattern of (**a**) Fe_3_C carbide after 1 h of tempering at 550 °C; (**b**) Cr_7_C_3_ carbide after 16 h of tempering at 600 °C; (**c**) and (**d**) Cr_23_C_6_ carbides after 16 h of tempering at 600 °C.

M_3_C carbides, mostly rich in Fe but also with Cr and Mo, exist in the matrix and also precipitate due to retained austenite decomposition. These M_3_C carbides dissolve and Cr_7_C_3_ and Cr_23_C_6_ carbides form [19]. Cr_23_C_6_ is the equilibrium carbide and a plateau is observed in the dilatometry curves after precipitation of this carbide. However, in order to determine the nature and morphology of carbides after tempering of the investigated steel, a TEM study was carried out. To this end, two samples were selected: One from the early stages of tempering, after 1 h at 550 °C, and the other, after 16 h of tempering at 600 °C in the middle of the carbides coarsening stage; results are reported in Figure 9.

As can be seen in Figure 9a, after 1 h of tempering at 550 °C, many carbides with various sizes are present in the microstructure. EDS (Energy-dispersive X-ray spectroscopy) analysis of the carbides in the carbon replica, revealed that they are very rich in Fe with nearly no presence of Cr. Hence, there is no chromium carbide formed during the first hour of tempering or they are very small to be extracted with carbon replicas. Figure 9a shows an F_3_C carbide with its related selected area electron diffraction (SAED) pattern at this tempering condition. No specific orientation between carbides and matrix was revealed in this study since all the TEM investigations were made on carbon extraction replicas. 

By increasing the time and temperature of tempering to 16 h at 600 °C, carbides tend to be richer in chromium. The carbide pointed by an arrow in Figure 9b is a Cr_7_C_3_ carbide based on SAED pattern, with rhombus-shape, orthorhombic crystal system and an equivalent diameter of 98 nm. As can be seen in Figure 9c, the other type of carbide detected by TEM and SAED is Cr_23_C_6_ with a cubic crystal system, which has a rod-like shape with an equivalent diameter of 95 nm; however, the coarser globular shape of Cr_23_C_6_ with an equivalent diameter of 404 nm can be observed as well. Therefore, the rod-like and spherical carbides that can be seen in SEM image of Figure 8c can be considered as Cr_23_C_6_ carbides.

Generally, carbides can be classified into four types according to their shape: needle-like, rod-like, lenticular and rhombus or globular. Table 2 summarizes the morphology, size and crystal structure of carbides that were identified in the investigated steel. It is interesting to note, while the obtained results are consistent with the observations made by several authors on Cr-Mo steels [19,31,32], in our study, no molybdenum carbide was identified in the material even after 16 h of tempering at 600 °C.

## 4. Conclusions

Retained austenite decomposition and carbides precipitation were investigated during isothermal tempering at 350 °C, 550 °C and 600 °C in a medium-carbon low-alloy bainitic steel. The following conclusions can be derived from the present study:

(1) Retained austenite decomposition starts at 350 °C but cannot be fully decomposed at this temperature nor at 550 °C. The final product of retained austenite decomposition depends on the tempering temperature. Undecomposed retained austenite remains in the microstructure during cooling from 350 °C, which requires further tempering at higher temperatures. In the case of 550 °C, fresh martensite forms from the undecomposed retained austenite during cooling. However, rising the tempering temperature to 600 °C led to a full decomposition of retained austenite during isothermal tempering.

(2) Dilatometry analyses of isothermal tempering curves at 550 °C showed that the decomposition of retained austenite only occurred during the first hour of tempering. Chromium carbide precipitation accompanied with hardening occurred at about 11.4 h of tempering at 550 °C. This precipitation time dropped to 4.7 h by increasing the temperature to 600 °C.

(3) No chromium carbide precipitated during the first hour of tempering at 550 °C. The Fe rich carbides were transformed to rhombus-shape Cr_7_C_3_, as well as rod-shape and globular Cr_23_C_6_ precipitated from the matrix during tempering at 600 °C, but no molybdenum carbide was observed.

## Figures and Tables

**Table 1:** Chemical composition of the investigated steel (wt.%).

C	Mn	Si	Ni	Cr	Mo	V	Cu
0.35	0.99	0.41	0.50	1.86	0.53	0.15	0.16

**Table 2:** Crystal structure, morphology and size of identified carbides in the tempered medium-carbon low-alloy bainite.

Carbide	Tempering Treatment	Crystal System	Morphology	Equivalent Diameter
Fe_3_C	1 h at 550 °C	Orthorhombic a = 5.092 Å, b = 6.741 Å, c= 4.527 Å	lenticular	-
Cr_7_C_3_	16 h at 600 °C	Orthorhombic a = 7.01 Å, b = 12.142 Å, c = 4.526 Å	rhombus-shape	98 nm
Cr_23_C_6_	16 h at 600 °C	Cubic a = 10.65 Å	rod-like globular	95 nm 404 nm
